# Activity-Aware Wearable System for Power-Efficient Prediction of Physiological Responses

**DOI:** 10.3390/s19030441

**Published:** 2019-01-22

**Authors:** Nathan Starliper, Farrokh Mohammadzadeh, Tanner Songkakul, Michelle Hernandez, Alper Bozkurt, Edgar Lobaton

**Affiliations:** 1 Department of Electrical and Computer Engineering, North Carolina State University, Raleigh, NC 27695, USA; nstarli@ncsu.edu (N.S.); ffmohamm@ncsu.edu (F.M.); tpsongka@ncsu.edu (T.S.); aybozkur@ncsu.edu (A.B.); 2 Department of Pediatrics, University of North Carolina School of Medicine, Chapel Hill, NC 27516, USA; michelle_hernandez@med.unc.edu

**Keywords:** wearable health, physiological prediction, activity clustering, multi-modal data, Body Sensor Networks, sensor selection, power efficient sensing

## Abstract

Wearable health monitoring has emerged as a promising solution to the growing need for remote health assessment and growing demand for personalized preventative care and wellness management. Vital signs can be monitored and alerts can be made when anomalies are detected, potentially improving patient outcomes. One major challenge for the use of wearable health devices is their energy efficiency and battery-lifetime, which motivates the recent efforts towards the development of self-powered wearable devices. This article proposes a method for context aware dynamic sensor selection for power optimized physiological prediction using multi-modal wearable data streams. We first cluster the data by physical activity using the accelerometer data, and then fit a group lasso model to each activity cluster. We find the optimal reduced set of groups of sensor features, in turn reducing power usage by duty cycling these and optimizing prediction accuracy. We show that using activity state-based contextual information increases accuracy while decreasing power usage. We also show that the reduced feature set can be used in other regression models increasing accuracy and decreasing energy burden. We demonstrate the potential reduction in power usage using a custom-designed multi-modal wearable system prototype.

## 1. Introduction

Body Sensor Networks and wearable health monitoring in particular have seen an unprecedented spike in interest over the past two decades [1,2,3]. These devices show promise in offering solutions in remote health monitoring and general wellness tracking to deal with the rapidly aging population and the associated increasing health care costs and financial burden. Application areas of wearable health monitoring include telehealth, telehealthcare, telemedicine, telecare, telehomecare, e-health, p-health, m-health, assistive technology, and gerontechnology [4,5]. For example, Kroll et al. [6] recently used wearables for remote health monitoring of post-discharge intensive care unit patients, with high detection rates of tachycardia (98.8%). With the rapid progress of sensor and wireless technologies, wearable health monitoring has become more accessible to the general population. This along with the emergent “Quantified Self” movement has lead to massive growth in popularity of smart-phone connected wearable consumer products and crowd-sourced health care and personalized fitness [7]. These devices have the potential to enable personalized preventative care leading to improved healthcare outcomes through early detection of vital sign anomalies in patients with chronic life-threatening conditions such as atrial fibrillation, epilepsy, or severe asthma. Wearable and implantable devices have been used in various medical applications from electrocardiogram (ECG) monitoring to fall detection. Many systems for ECG monitoring using wearable sensors and mobile phones have recently been developed [8,9,10]. Non-invasive wearable sensors are also being developed for glucose monitoring in diabetic patients [11]. Other devices have focused on general wellness and personalized health tracking [12] and related applications such as perspiration analysis and monitoring [13]. One of the biggest applications of wearable devices is in human activity monitoring [14]. Researchers have used activity monitoring for analyzing the gait and balance of Alzheimer’s [15] and Parkinson’s [16,17,18] patients, fall detection in elderly patients [19,20,21], and rehabilitation [22]. Wearable sensors have also been used extensively in applications other than health monitoring such as safety monitoring on construction sites [23].

One of the major challenges to the development and widespread adoption of wearable health is the energy requirements of these devices. Energy requirements will continue to rise as the computational capabilities and functionality of these devices become more advanced [24]. This poses a problem as extended battery life is critical in remote health applications where constant vital sign monitoring is required. In addition, frequent charging makes the user experience less convenient and increases the likelihood that users will either use the devices incorrectly or stop using them altogether. Wearable devices also must be small and lightweight to prevent being intrusive to the user’s lifestyle. Therefore, they should have small batteries, further limiting the energy available to these devices. Many health and wellness monitoring platforms are comprised of multiple sensing modalities for monitoring numerous vital signs, further increasing the energy burden. In the case of implantable sensors, charging is even more challenging and therefore an extended battery life is even more critical. Major research focus areas for energy efficiency include low power transmission, low power electronics, energy harvesting, and adaptive energy efficient sampling and sensor selection techniques [24]. Low power and self-powered devices are a particularly popular area of research currently with many devices under development [25,26]. Many recent advances in flexible triboelectric and piezoelectric nanogenerators are making the development of fully self-powered wearable and implantable sensors more achievable [27,28,29,30]. For example, Lin et al. [31] recently developed a self-powered HR monitoring BSN enabled by triboelectric nanogenerators. However, current self-powered devices still face numerous challenges before complete energy autonomy is achievable, including improving energy density, flexibility and conformability, and energy management circuits [26]. Therefore, other techniques to reduce energy requirements such as energy efficient adaptive sampling and sensor selection techniques could potentially improve the performance and viability of self-powered devices.

**Figure 1:**
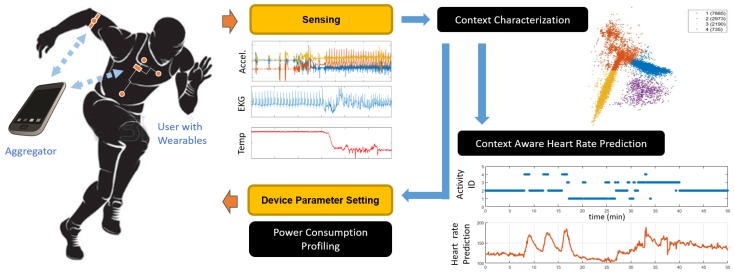
Overview of the proposed pipeline for sensor selection and activity aware prediction. Multimodal sensor information is sensed by the wearable system and transmitted to an aggregator for fusion and prediction. Context information is used for physiological response prediction and sensor selection by disabling certain modalities, and hence reduce power consumption.

In this article, we focus on a context-aware dynamic sensor selection method for joint optimization of power efficiency and heart rate (HR) prediction accuracy in a multi-modal wearable health platform. The wearable platform consists of multiple sensor streams including body and environmental temperature, heart rate (HR), humidity, accelerometer based activity levels of wrist and ankle, and electrodermal activity (EDA). Various temporal and spectral features are extracted from each data stream. We use a dynamic feature selection approach based on contextual awareness of physical activity from the accelerometer data. We first cluster the data into different activities and then perform a group lasso regression for HR prediction which reduces the set of grouped features representing the various sensors while optimizing prediction accuracy. This allows us to activate and deactivate the relevant sensors depending on current activity cluster and in turn reduce power usage. Figure 1 illustrates this process. The HR predicted using contextual awareness could be compared to the actual measured HR for anomaly detection, for example, bradycardia or tachycardia. Unfortunately, we cannot measure the power levels on this closed device systems. To overcome this issue, we collect power data on the Health and Environment Tracker (HET), a lower power multiparameter wearable health device under development at the National Science Foundation Nanosystems Engineering Research Center for Advanced Self-Powered Systems of Integrated Sensors and Technologies (ASSIST Center) [32]. The details of this system are presented in Section 3.2. On the HET devices, we show the effect of selecting specific sensors determined through our method on total power usage. Although we focus on HR prediction in this paper, this method could be applied to numerous other prediction scenarios using wearable sensors depending on the need of the health application. The main contributions of this work include:

1. Providing preliminary results and demonstrating the benefits of our activity-aware and personalized prediction of physiological signals
2. Investigating the trade-off between accuracy and power usage using our methodology
3. Demonstrating the power reduction due to our methodology in a real wearable platform

Our study shows promising results for both prediction and power reduction. Our sensor selection method reduces power consumption of the wearable system by up to 38%. We show that, while prediction accuracy increases slightly with activity clustering, power usage is reduced drastically. We also show that we are able to increase prediction accuracy and reduce power usage using the reduced set of sensors determined by the group lasso regularization on another regression model, namely a Support Vector Machine (SVM) with Gaussian kernel, demonstrating the potential to use our reduced set of features in other models. We investigated the trade-off between prediction accuracy and power usage, showing that the power can be further reduced if we can accept a minor decrease in accuracy.

The rest of the article is organized as follows. In Section 2, we provide the background for the various sensor streams we use in our prediction model as well as some related work in physiological prediction and power efficiency in wearable systems. In Section 3, we discuss the sensors used and the data collection protocol followed. In Section 3.2, we describe the prototype multimodal wearable device we use for demonstrating power reduction using our method. In Section 4, we give the detailed mathematical formulation of our problem, methodology, technique, and evaluation. Finally, we discuss our experimental results in Section 5 for prediction performance and power reduction, and discuss the trade-off between power efficiency and prediction accuracy using our method.

## 2. Background and Related Work

In this study, we investigated the relationship among physiological signals, environmental conditions, and activity level. Our method builds a linear model for HR where as predictors we extract various features from measurements of environmental temperature and humidity, body temperature, ankle acceleration, wrist acceleration, and EDA. The relationship between many of these signals in controlled settings have been studied in depth in the literature. The effect of exercise and heat stress on cardiovascular response is a well known relationship [33] as well as the effects of air temperature and humidity [34]. It is also well known that HR and EDA are indicators of psychological and physiological arousal [35]. Few studies have investigated using physical activity to predict HR. For example, Xiao et al. [36] used an evolutionary neural network to predict HR from physical activity. However, to the best knowledge of the authors, there is no study that incorporates the activity information and all the physiological signals considered in this study for prediction of HR in a generic exercising scenarios with an aim of using it for energy efficiency improvement.

The idea behind our model is to continuously monitor users and predict expected “normal” behavior of vital signs based on contextual information. Research has shown that continuous monitoring using a wearable system would provide potential benefits to self-management of conditions during daily life. For example, adolescents and young adults poorly perceive their asthma symptoms [37,38,39,40]. Poor symptom perception can reduce a young person’s ability to recognize when their symptoms are worsening, thereby decreasing the probability that they would take prophylactic action that could prevent further symptom progression and subsequent exacerbations. Indeed, the negative impacts of asthma are largely preventable if young adults engage in self-management behaviors, including symptom monitoring [41,42,43]. Monitoring measurable physiologic changes using wearable sensor-based systems would increase the ability of young adults to identify an impending exacerbation, prompting them to proactively start rescue therapy and reduce the risk of progressing to exacerbation. These expected target values could be compared to actual measured values to warn users of vital sign anomalies associated with an impending asthma exacerbation. A similar argument can be made for other chronic conditions such as heart diseases.

Many studies have investigated the monitoring of physiological data for detection of anomalies [3,44]. In particular, Clifton et al. [45] used Gaussian Processes to model and predict arterial oxygen saturation for the detection of a deterioration event in hospital patients. However, this approach is geared towards prediction in a more steady state activity profile (i.e., while sedentary in a hospital-based setting) without accounting for motion artifacts encountered during routine activities of daily living. Our research aimed to develop accurate personalized models that adapt to changing activity states in a broader patient population.

There has been much research into improving the power efficiency of wearable health systems through adaptive sampling and similar sensor selection strategies [46]. French et al. [47] used selective sampling to conserve power in context recognition wearable systems. They investigated the effects of sampling techniques on activity recognition accuracy and power reduction. Other studies use sensor selection strategies to successfully decrease power consumption [48,49]. Our method aims to select the optimal set of features that not only reduces power consumption but also maximizes prediction accuracy by making more informed context-aware decisions based on activity identification.

## 3. Data Collection

**Figure 2:**
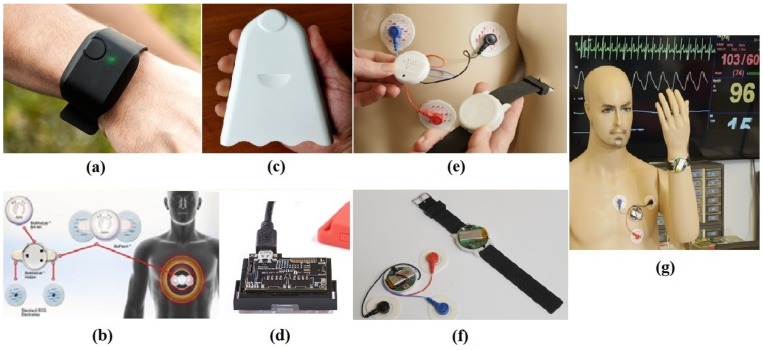
(**a**) E4 wristband; (**b**) BioHarness electrode placement on chest; (**c**) Airbeam module; (**d**) SensorTag; (**e**) HET chest electrode placement; (**f**) HET chest patch and wristband; and (**g**) HET chest patch and wristband as worn on the body.

In this section, we describe the wearable health monitoring platforms (Section 3.1 and Section 3.2) and the protocol (Section 3.3) used for data collection. Figure 2a–d shows the main devices used. The active sensors were ambient temperature, relative humidity, accelerometer based measurement of activity at the wrist and ankle, body temperature measured at the skin, electrodermal activity and electrocardiogram (EKG) based HR. To be able to let others reproduce our prediction results and have access to our data, we collected the sensor data using commercially off-the-shelf wearables as listed below. However, as it is difficult to make power measurements on these closed and proprietary devices, for the later part of our study, we used the ASSIST HET devices where we had access to all the nodes on the electronics to be able to make measurements on hardware. As the ASSIST devices also used commercial off the shelf integrated circuits and discrete sensor chips, the recorded current numbers are still relevant and would reflect the power trends in such commercial wearables. All data collected in this study are available online [50].

### 3.1. Off-The-Shelf Devices

- **Aircasting AirBeam (by HabitatMap LLC, New York, NY, USA)** [51]. We used this portable air monitor to measure relative humidity and ambient temperature with a sampling rate of 1 Hz.
- **BioHarness (by BIOPAC Systems Inc., Goleta, CA, USA)** [52]. This lightweight portable device developed by BIOPAC measures EKG based HR. The BioHarness is capable of recording data accurately even when the subject is performing vigorous activities or exercise. It is comprised of a chest strap incorporated in a sports shirt and a separable measurement module affixed to the strap. BioHarness measurements of HR were experimentally validated by showing their strong relationship to actual, reliable values (r=0.89 to 0.99, p<0.01) [53]. In this study, we used HR sampled at 1 Hz.
- **E4 (Empatica Inc., Cambridge, MA, USA)** [54]. This wearable wristband offers real time data acquisition for various physiological responses. The E4 contains an EDA sensor that constantly measures sympathetic nervous system arousal sampled at 4 Hz. EDA data include information about the user’s stress, engagement, and excitement level. A three-axis accelerometer with sampling rate of 32 Hz and an infrared thermopile, measuring peripheral skin temperature sampled at 4 Hz, are also incorporated in the E4 wristband. We used body temperature, acceleration of the wrist, and EDA as measured by this device.
- **SensorTag (Texas Instruments, Dallas, TX, USA)** [55]. This device is a low power development board by Texas Instruments containing an accelerometer and gyroscope. These data were sampled at a rate of 100 Hz.

### 3.2. HET Wearable Platform

In this section, we discuss the prototype system we used for quantifying the power savings of our method in a wearable device. Under the ASSIST Center, the Health and Environmental Tracker (HET) system is developed to demonstrate core technologies for achieving a high-performance and multi-functional biomedical sensing system with minimized power consumed via low-power sensing, subthreshold transistor computation, novel transistor designs, and ultra-low power radios. The ultimate goal for this device is to realize sufficiently low power consumption such that the system could utilize continuous energy harvesting to achieve self-powered operation. This system was evaluated as a wireless ultra-low power system for assessing multimodal sensing of environmental and health parameters and tested on human subjects [25,56]. The system is also accessible to ASSIST Center research partners.

For this study, we used a version of the HET system that includes a wristband and chestpatch consisting of relevant sensors to this study. The wristband sensors can track wrist motion using an accelerometer, ambient temperature and humidity levels. The chest patch sensors are capable of measuring EKG based HR. To handle the amount of data being generated by these wearable devices, the system constantly transmits the recordings to a Bluetooth Low Energy (BLE) enabled peripheral data aggregation device (e.g., a laptop, tablet computer, or smartphone). Both the HET wristband and chest patch are controlled by a CC2541 system-on-chip (Texas Instruments, Dallas, TX, USA), which communicates with various sensors and transmits sampled data via BLE. Sampling is managed via a real-time operating system, allowing for different sampling rates for different sensors. Low-power modes are used to reduce power consumed while the system is idle between samples. Table 1 shows the integrated circuits and sampling rates utilized for the basic measurements taken by the HET wristband and chest patch. Both systems are powered by a rechargeable 3.7 V 450 mAh Lithium Polymer battery.

### 3.3. Data Acquisition Protocol

The data collection was undertaken at North Carolina State University under Institutional Review Board 7799. We recorded physiological activity and environmental data on a healthy, 25–30 year old male subject over a period of four separated days. The subject wore the aforementioned devices in the following manner: The E4 was worn on the left wrist, following the manufacturer’s instructions. The SensorTag was strapped to the right ankle to capture lower body movement. The BioHarness was worn using the provided sports shirt that contains the built-in chest strap. The AirBeam was carried on the subject’s back except during workout routines in which the device would be obstructive. During exercise, the AirBeam was kept close to the subject to measure his environment’s temperature and humidity.

Since the BioHarness and E4 keep track of time locally and the SensorTag and Airbeam stream directly to the cell phone, the subject was asked to jump at the beginning and at the end of each recording session. This event was captured by all recording devices and was used to synchronize all data channels. The temperature and humidity data streams share the internal time stamp of the cell phone with the SensorTag, therefore the SensorTag was used to synchronize those data streams. The subject started the data collection by walking outdoors for 10 min. Then, he alternated between walking one lap and running one lap on a 250 m long indoor track for 20 min. He then rested for 10 min and performed two 15 min long sessions of various exercises with 5 min of resting in between. The exercises include chest, leg, and abdominal workout routines established by a third party. Next, the subject walked indoors for 10 min and then left the building and walked outdoors for another 10 min. The main intention of the protocol was to obtain highly varying HR data during various activities in an uncontrolled environment. The same data collection routine was repeated in the remaining three days.

We collected measurements of average current draw of the HET device and the effect of deactivating various sensors. Here, deactivating means placing the sensor in idle mode and stopping all Bluetooth communications for that modality. Table 2 gives current draws for various operating states: all sensors active and transmitting, and current draw added when individual sensors were disabled. These were the values used in our study to estimate power reduction. We used the HET chest accelerometer power calculations for the TI SensorTag accelerometer values (worn on the ankle). We also assumed for simplicity that the skin temperature and EDA sensors draw the same current as the wrist accelerometer based on the data sheet comparisons.

## 4. Methodology

In this section, we introduce our framework (Section 4.1) for context-aware prediction of physiological signals, our approach to context identification based on activity recognition (Section 4.2), the specific method used for prediction and sensor selection (Section 4.3), and the evaluation metrics used for the analysis (Section 4.4).

### 4.1. Mathematical Formulation

Let us consider a set of sensor streams ${\left\{x_{k}\left(t\right)\right\}}_{k=1}^{N_{s}}$ where *k* is the sensor index, $N_{s}$ is the number of sensors, $x_{k}\left(t\right)\in R^{d_{k}}$, and $d_{k}$ is the dimensionality of the stream (e.g., for temperature $d_{k}=1$ and for accelerometers $d_{k}=3$). Each sensor stream is associated with a corresponding sensor device and has an average power consumption $P_{k}$. Let $X\left(t\right)=[x_{1}\left(t\right),\cdots ,x_{N_{s}}\left(t\right)]$ be the full data stream, and let the subset of sensor streams used for context-awareness (based on the activity state) be given by $X_{a}\left(t\right)=\left[x_{i_{1}}\left(t\right),\cdots ,x_{i_{N_{a}}}\left(t\right)\right]$ where $I_{a}=\left\{i_{1},\cdots ,i_{N_{a}}\right\}$ is the corresponding index set of sensor streams used for context-awareness, and $N_{a}$ is the number of sensors used for this purpose. Furthermore, we let $y\left(t\right)\in R^{d}$ be the target physiological stream that we aim to predict. We focus on the analysis of HR prediction for which d=1.

We define the activity cluster function

(1) $$C\left(t\right)=f\left(X_{a}\left(\left(-\infty ,t\right]\right),t\right)$$

which maps sensor streams to the corresponding activity index {1,⋯,K}. The notation $X_{a}\left(E\right)$ indicates that all past values of $X_{a}$ in that interval *E* are utilized for context-awareness. In our implementation, we only consider features extracted over a fixed time window, but this can be easily generalized to incorporate time-series models or recurring neural networks that incorporate longer temporal dependencies. For each cluster index *c*, we define a prediction model of the form

(2) $${\hat{y}}_{c}\left(t\right)=g_{c}\left(X\left(\left(-\infty ,t\right]\right),y\left(\left(-\infty ,t-\delta_{h}\right]\right),t\right),$$

where $\delta_{h}$ is a hyper-parameter which depends on the specific application and y(E) indicates that all past values of *y* in the interval *E* are utilized. The final prediction for our approach is given by $\hat{y}\left(t\right)={\hat{y}}_{c}\left(t\right)$ where c=C(t).

Ideally, we would predict a normal physiological response based only on other physiological, environmental and motion-based characteristics (i.e., $\delta_{h}=\infty$). An anomaly detection could then be associated to a large deviation from the normal response (i.e., if $||y\left(t\right)-{\hat{y}}_{c}\left(t\right)\left|\right|$ is large enough). However, as we present in Section 5, the predictive power drops drastically for larger values of $\delta_{h}$. Hence, we chose to include some of the past values of the target stream. The choice of $\delta_{h}$ should be based on the expected time response constant for the anomaly. For example, if an anomaly in HR is expected to become significant after 30 s (e.g., during the first 30 s there is a slow increase on HR leading to a noticeable deviation from a normal response after that period), then we can use $\delta_{h}=30$ to detect this anomaly. Clearly, if we choose a $\delta_{h}$ that is too small, then we may not be able to detect any anomalies if they are not expected to be significant within that time window.

Additionally, we were interested in reducing the average power consumption of our system. This reduction in power was driven by enforcing sparsity in the context-aware prediction models, as presented in Section 4.3.

### 4.2. Activity State Recognition

Our current research aimed to explore the effect of activity recognition on prediction accuracy and power reduction. We believe that the number and type of sensors required vary among activity states. As described in Section 4.1, we aimed to develop separate regularized regression models for each activity state allowing the use of fewer sensors during specific activities and therefore reducing total power consumption and increasing prediction performance by removing irrelevant features. There is a very large amount of research on activity recognition using wearable sensors [57]. Many papers detail recognition accuracy using various sets of time and frequency domain features [57,58,59,60]. Most state-of-the-art feature extraction techniques use a sliding window based approach with a sliding window time length anywhere from 0.25 to 30 s [57], with recent studies showing a good trade-off between accuracy and computational burden around the 1–5 s range [61,62].

**Figure 3:**
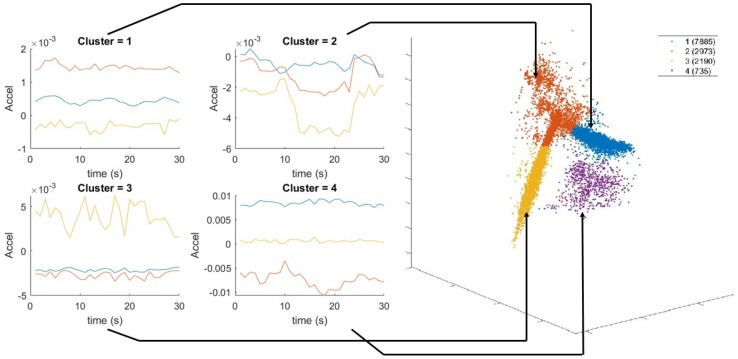
Example 30 s windows of accelerometer data after LPP reduction corresponding to each respective activity cluster. The orange, blue, and yellow curves represent the time series of the three dimensions of the accelerometer data after dimensionality was reduced from 21 to 3.

We performed activity recognition using the ankle accelerometer, however other locations can be used as well. The features extracted using a 5 s sliding window are the mean of accelerometer and gyroscope; and variance, covariance, skewness, kurtosis, peak spectral magnitude, and corresponding peak frequency for each accelerometer axis. We reduced the feature space from 21 to 3 using locality preserving projections (LPP) [63]. We then performed k-means clustering [64] with k=4 to partition the data. The number of clusters was selected to match the main four activity states present during data collection (i.e., walking, running, resting, and exercising). Unsupervised clustering was chosen to most accurately reflect the uncontrolled scenarios in which labeling activities for training for each person is cumbersome and introduces a possible source of user error. The training data clusters are shown in Figure 3. Based on visual inspection, we concluded that Clusters 1, 2, 3 and 4 correspond mainly to walking, weightlifting, resting and running, respectively.

### 4.3. HR Prediction

In this section, we briefly describe our prediction model based on $L_{1}$ regularization for linear regression which is the most commonly used form of lasso. This provides a realization of the $g_{c}\left(\cdot ,\cdot ,\cdot \right)$ prediction model defined in Section 4.1. Consider a dataset with *N* observations and *p* predictors, where the response vector is $Y={\left[y_{1},\cdots ,y_{N}\right]}^{⊤}$ and the feature matrix is $Z=[z_{1}\left|\cdots \right|z_{p}]\in R^{N\times p}$ formed from the predictor vectors $z_{j}={\left[z_{j,1},\cdots ,z_{j,N}\right]}^{⊤}$. In our application, the predictor vectors $z_{j}$ correspond to the temporal and spectral features (see details below in this section) extracted from the sensor streams X(−∞,t) and the historic values of $y(-\infty ,t-\delta_{h})$, the approach aims to predict the response variable *y* at time *t*.

The common linear regression model is represented by Y=Zβ+ϵ, where $\beta ={\left[\beta_{1},\cdots ,\beta_{p}\right]}^{⊤}$ is the vector of linear predictor coefficients. Group lasso was proposed in [65] to include or exclude predetermined groups of features while finding an optimal prediction model. The formalization of this approach is a generalized form of standard lasso:

(3) $$\hat{\beta }=\underset{\beta }{\mathrm{argmin}}\;{∥Y-\sum_{k=1}^{N_{g}}Z_{k}\beta_{k}∥}_{2}^{2}+\lambda \sum_{k=1}^{N_{g}}{∥\beta_{k}∥}_{W_{k}},$$

where the feature matrix Z has been partitioned into $N_{g}$ groups ${\left\{Z_{k}\right\}}_{k=1}^{N_{g}}$ with corresponding coefficients ${\left\{\beta_{k}\right\}}_{k=1}^{N_{g}}$, ${\left\{W_{j}\right\}}_{k=1}^{N_{g}}$ are positive definite matrices, and ${∥\eta ∥}_{W}={\left(\eta^{\prime }W\eta \right)}^{1/2}$. If each group contains only one feature with $W_{k}=1$, then Equation (3) reduces to the standard lasso. On the other hand, if all the features are in one group, it reduces to ridge regression. The weight λ determines the sparsity of the model. A small value of λ yields a solution similar to the least squares estimation. By increasing the value of λ, the model is forced to set more and more coefficients of each group equal to zero. This parameter can be used to manage the number of groups included in the regression process.

In our case, the matrices $Z_{k}$ and vectors $\beta_{k}$ represent the features and coefficients for each sensor modality, and $N_{g}=N_{s}$ (i.e., the number of sensors). All predictors and responses are normalized to have zero mean and standard deviation of one before processing through the model. We chose $W_{k}=\frac{1}{\sqrt{M_{k}}}I_{M_{k}}$, where $M_{k}$ is the number of features in group *k* and $I_{M_{k}}$ is the identity matrix of size $M_{k}$. We refer to this problem as the normalized approach. This choice weights each group equally based on the root mean square of $\beta_{k}$ and does not penalize for having more features extracted from a sensor. In our setup, the features are extracted in the aggregator and therefore do not affect power consumption of the wearable devices. We also considered the case when $W_{k}=\frac{P_{k}}{\sqrt{M_{k}}}I_{M_{k}}$, where each group is weighted by the average power consumption $P_{k}$ for each sensor. We refer to this problem as the weighted approach. Note that, although we only used a few devices, each device has multiple sensors. For instance, the E4 device contains three separate feature groups, namely one group for each EDA, accelerometer, and skin temperature sensor. Hence, $N_{s}=7$ in our application. The seven feature groups include: body temperature, environmental temperature, humidity, wrist acceleration, ankle acceleration, EDA, and HR. The first three groups are single feature groups using the raw values of each measurement as features. The wrist and ankle accelerometer feature groups consist of the 21 features extracted as described previously. The EDA feature group consists of six features: mean, linear trend, variance, skewness, kurtosis, and absolute range over a sliding window. Finally the HR feature group consists of three features namely, the HR and the first and second differences of the hear rate signal. The motivation for the accelerometer features we chose is discussed in Section 4.2 while the EDA features are motivated by previous studies [66,67].

### 4.4. Evaluation Metrics

We performed cross-validation (CV) to select the optimal value for our regularization term λ separately on each cluster in order to build activity-dependent models. We split the data into training, validation, and testing across days. We used Days 1, 2, and 3 as training data and Day 4 as our test data. We performed three-fold cross-validation on the three training days by permuting days as folds. We chose to split the data into folds corresponding to the days in order to ensure that all folds include data from all activity clusters. We selected the value for λ that minimizes the average root mean square error (RMSE) across folds.

We evaluated our prediction model on the test set using the following metrics: mean absolute error (MAE), root mean square error (RMSE), normalized root mean square error (NRMSE), and coefficient of determination ($R^{2}$). They are defined as:

(4) $$MAE=\frac{1}{N}\sum_{i=1}^{N}{\left|{\hat{y}}_{i}-y_{i}\right|}^{2}$$

(5) $$RMSE=\sum_{i=1}^{N}\sqrt{\frac{1}{N}{\left({\hat{y}}_{i}-y_{i}\right)}^{2}}$$

(6) $$NRMSE=\sum_{i=1}^{N}\frac{\sqrt{\frac{1}{N}{\left(y_{i}-{\hat{y}}_{i}\right)}^{2}}}{\left(y_{max}-y_{min}\right)}$$

(7) $$R^{2}=1-\frac{\sum_{i=1}^{N}{\left({\hat{y}}_{i}-y_{i}\right)}^{2}}{\sum_{i=1}^{N}{\left(y_{i}-\bar{y}\right)}^{2}}$$

where $\left\{y_{1},\cdots ,y_{N}\right\}$ are the ground truth response values; $\bar{y}$, $y_{max}$ and $y_{min}$ are the mean, maximum, and minimum values of the response variables, respectively; $\left\{{\hat{y}}_{1},\cdots ,{\hat{y}}_{N}\right\}$ are the predicted values; and *N* is the number of test points.

We evaluated our power reduction using average current reduction per cluster, $I_{c_{i}}$, and total average current reduction across clusters:

(8) $$I_{avg}=\frac{\sum_{i}\left(N_{c_{i}}\cdot I_{c_{i}}\right)}{\sum_{i}N_{c_{i}}}$$

where $N_{c_{i}}$ represents the number of points in cluster *i*.

## 5. Results and Discussion

**Figure 4:**
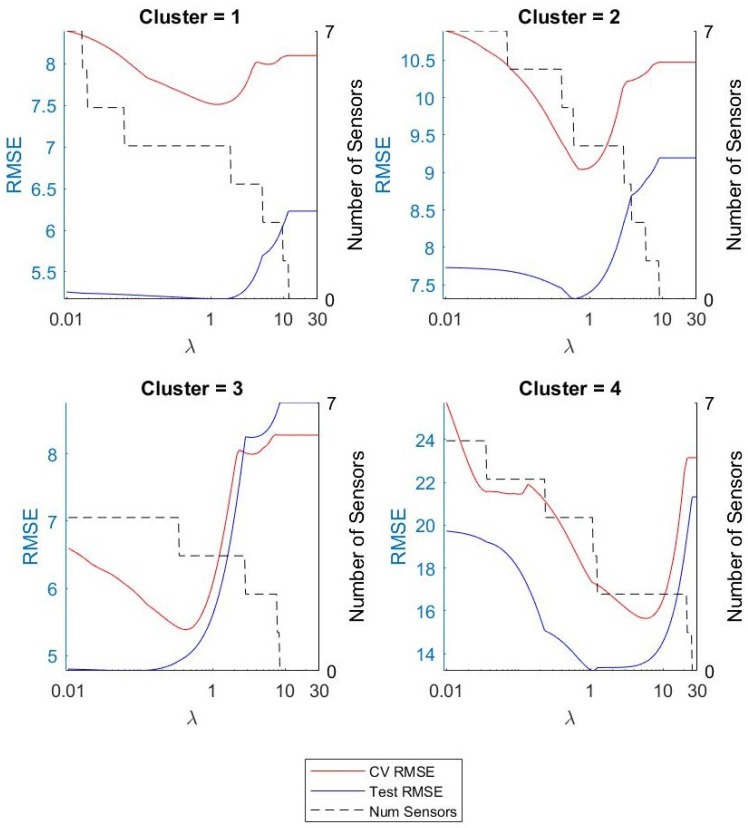
RMSE and number of sensors used versus λ for each cluster.

We constructed models for each of the activity clusters for $\delta_{h}=$ 10, 30, 60, 90, and *∞*, where $\delta_{h}=\infty$ refers to no past HR features being used as predictors. We did the same for the model without utilizing activity clustering (one model for all data). We studied the effect of $\delta_{h}$, activity clustering, and feature selection on prediction accuracy and power efficiency. We selected the value for our regularization term λ using cross validation as described previously. Figure 4 shows the results for $\delta_{h}=30$. The figure shows the average RMSE across CV folds, final RMSE on the test data, and the number of sensors included in the model as a function of λ. As was expected, the RMSE decreased as λ increased and we removed irrelevant features until we found a minimum RMSE, which gave us our optimal value. Then, as we increased λ, we began to remove relevant predictors from the model and saw an increase in error.

**Figure 5:**
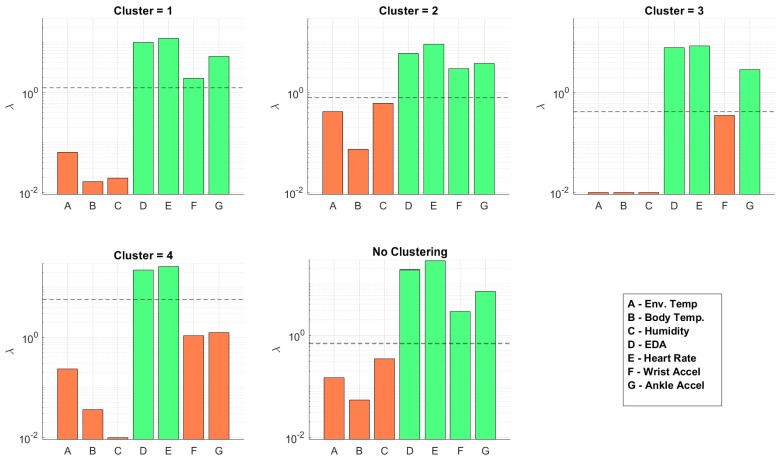
λ-levels at which sensors are excluded and included in the model for each cluster as well as without clustering.

Figure 5 shows the λ-levels at which each sensor is removed from the model for $\delta_{h}=30$. The horizontal dotted line shows the optimal λ found through CV. An orange bar indicates the sensor was removed from the model and a green bar indicates the sensor was included with the optimal λ value. We show the sensor usage levels for all clusters and also when implementing the model without clustering. The figure shows that the number of sensors reduced with activity clustering. Table 3 lists the sensors included in the model for each cluster across all values of $\delta_{h}$. The most striking detail here is that the environmental sensors and body temperature were removed from all models. These features were fairly stable (i.e., there is not much variation in the dataset) and were mostly uncorrelated to the dynamic HR measurement compared to the accelerometer features. We can see that the wrist and ankle accelerometer sensors were the most important in all models. We notice this is particularly true in Cluster 4 (running). This is most likely due to the EDA and HR measurement being inaccurate and/or containing motion artifacts at higher activity levels (which is the case with most wearable EKG sensors). This could also be explained by the HR being highly dynamic during increased physical activity, therefore requiring highly dynamic features for prediction. We also notice that more sensors were required as we increased $\delta_{h}$. This was an expected outcome. As the past HR became less correlated with current HR, more features were required for prediction.

### 5.1. HR Prediction

**Figure 6:**
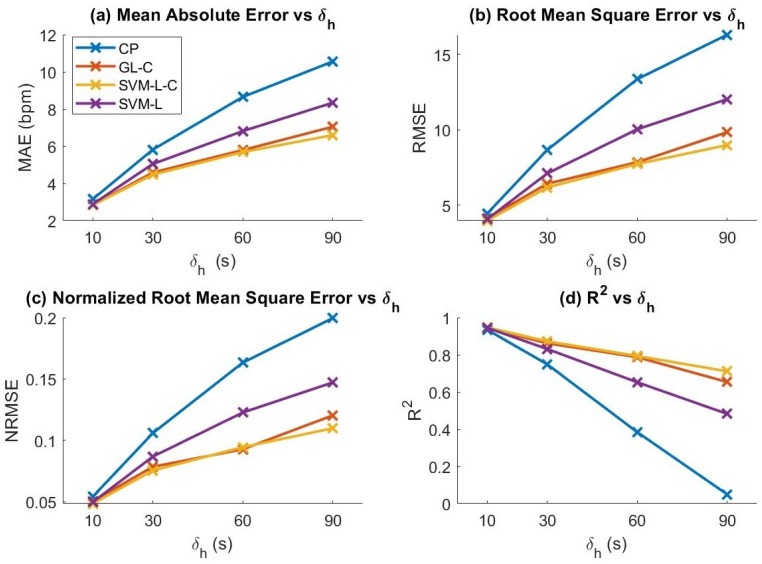
Prediction evaluation metrics of various models for increasing values of HR window history length ($\delta_{h}$). Evaluation metrics shown: (**a**) MAE; (**b**) RMSE; (**c**) NRMSE; and (**d**) $R^{2}$.

We evaluated the HR prediction accuracy of our models using the evaluation metrics defined in Section 4.4 (MAE, RMSE, NRMSE, and $R^{2}$). We evaluated our model with and without activity clustering. We also compared SVM performance with and without our activity clustering and feature set selection methodology. The models considered were: Group lasso with activity clustering (GL-C), group lasso without clustering (GL), support vector machine with GL-reduced feature set and clustering (SVM-L-C), SVM with reduced features without clustering (SVM-L), SVM with full feature set with clustering (SVM-C), and SVM with full feature set without clustering (SVM). We then compared our results to a constant predictor (CP), which uses the value of HR at time $t-\delta_{h}$ as the prediction. The results of the analysis are shown in Figure 6. We found that GL, SVM-C, and SVM achieved only slightly lower performance than GL-C, SVM-L-C, and SVM-L, respectively, thus these results were omitted from the figure for clarity. We observed that clustering only slightly enhances prediction performance for the group lasso models while substantially improving performance for the SVM models. In addition, reducing the dimensionality of the data using group lasso before feeding to the SVM model had very little effect on performance of SVM prediction; however, as we present in Section 5.2, it had a significant effect on power efficiency. We also observed that SVM outperformed the GL regression model, demonstrating the potential of our method to be applied to other regression/forecasting models.

We observed that, as we increased $\delta_{h}$, the performance of the constant predictor diverged more rapidly than the other models for all metrics. From this observation, we can infer that the other physiological and motion features contributed significantly to the prediction. However, we also found that the models did not perform significantly better than the cluster mean of the training data for $\delta_{h}=\infty$. We believe that this may be due to our limited dataset causing over-fitting of the model.

**Figure 7:**
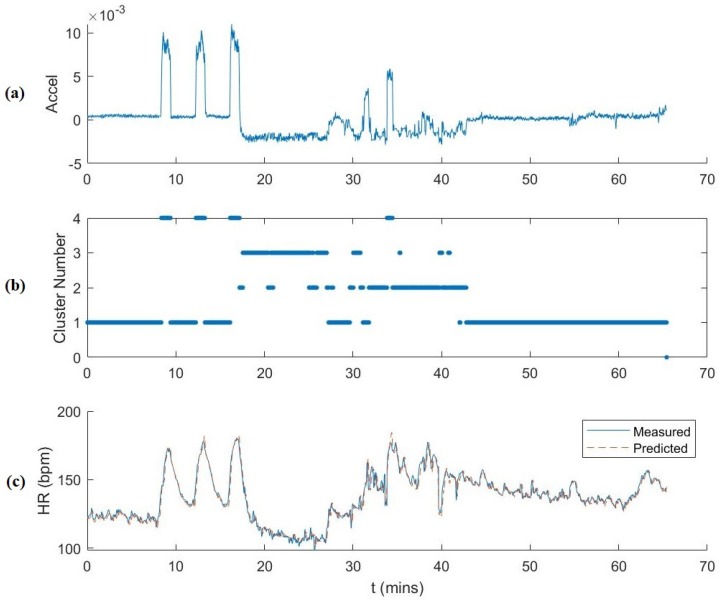
Illustration of the prediction process ($\delta_{h}=30$ s). From top to bottom: (**a**) the first dimension of the reduced accelerometer data features used for clustering over time; (**b**) activity cluster number over time; and (**c**) measured and predicted HR over time.

Figure 7 shows the full testing data plotted over time for $\delta_{h}=30$ s. The plots show the correlation between the first component of the 3D reduced dimensionality ankle accelerometer data, the cluster number, and the HR. HR prediction is also plotted. We observed that the accelerometer data accurately corresponded to activity cluster and thus a correlation with HR behavior as well. As mentioned previously, Clusters 1, 2, 3 and 4 correspond mainly to walking, workout, rest and running, respectively. The temporal plot of the activity clustering follows the experimental protocol. There were some cluster outliers during the workout sessions that were clustered as resting and walking. This is due to the dynamic movements of workouts and also because of rest periods between workout sets and the necessity of walking around to the various workout equipment during the session. We also observed that the majority of the large prediction residuals are during transitional periods between activities for which we do not have many observations.

### 5.2. HET Power Reduction

We used the measured current consumption values in Table 2 to determine the potential power savings using our sensor selection methodology. Table 4 shows the current reduction per cluster *i*, $\Delta I_{c_{i}}$, as well as the average total current reduction across all clusters, $\Delta I_{avg}$. We also show current usage without activity clustering to compare the effect of clustering on current consumption. The results show that activity clustering decreases the current consumption in all activity clusters as well as the total average current consumption for all values of $\delta_{h}$. As we expected, we could obtain major decreases in current consumption with very minor effect on the prediction accuracy. This information could be used to further personalize the models to specific individuals depending on the needs of the application.

### 5.3. Accuracy Power Trade-off

**Figure 8:**
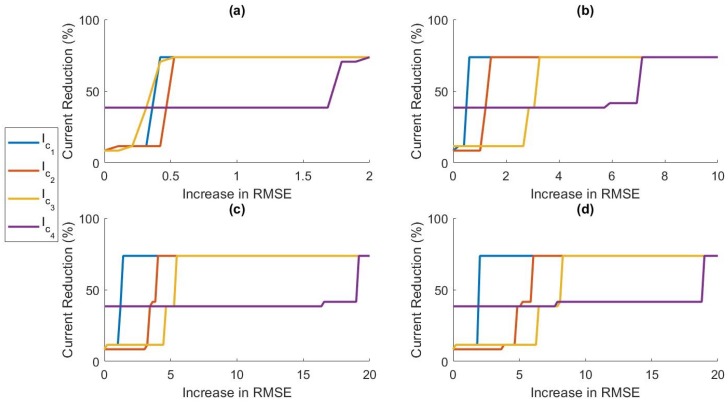
The effect of increasing allowable prediction RMSE on the current consumption for: (**a**) $\delta_{h}=10$; (**b**) $\delta_{h}=30$; (**c**) $\delta_{h}=60$; and (**d**) $\delta_{h}=90$, for each cluster.

**Figure 9:**
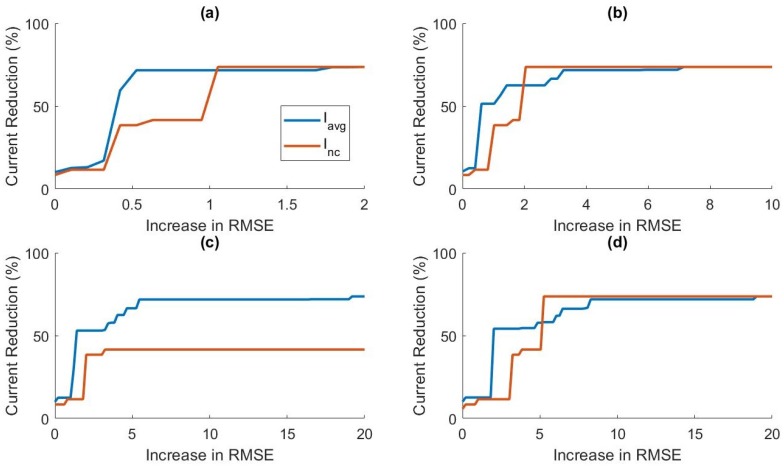
The effect of increasing allowable prediction RMSE on the average current consumption across clusters, $I_{avg}$, and no clustering, $I_{nc}$ for: (**a**) $\delta_{h}=10$; (**b**) $\delta_{h}=30$; (**c**) $\delta_{h}=60$; and (**d**) $\delta_{h}=90$.

In this section, we discuss the trade-off between prediction accuracy and power efficiency. In Figure 4, it can be observed that, as we increased λ past the optimal value, the RMSE began to increase while the number of sensors included in the model continued to decrease. In Figure 5, we can see that relatively small increases in λ past the optimal λ-level could remove more sensors from the model. This led us to believe that we could increase power savings even more by allowing small increases in prediction error. In applications where battery life is critical, small decreases in prediction accuracy may be preferable to higher current consumption. Figure 8 shows the relationship between RMSE increase and percent current reduction for each activity cluster for various values of $\delta_{h}$. Figure 9 compares the power tradeoff relationship between the average percent current reduction across clusters and the reduction in the model without clustering, $\Delta I_{NC}$. As hypothesized, significant increases in power reduction could be achieved with small increases in prediction RMSE. This information can be used to further customize the models to the specific application and patient need by adjusting the value of λ appropriately.

**Figure 10:**
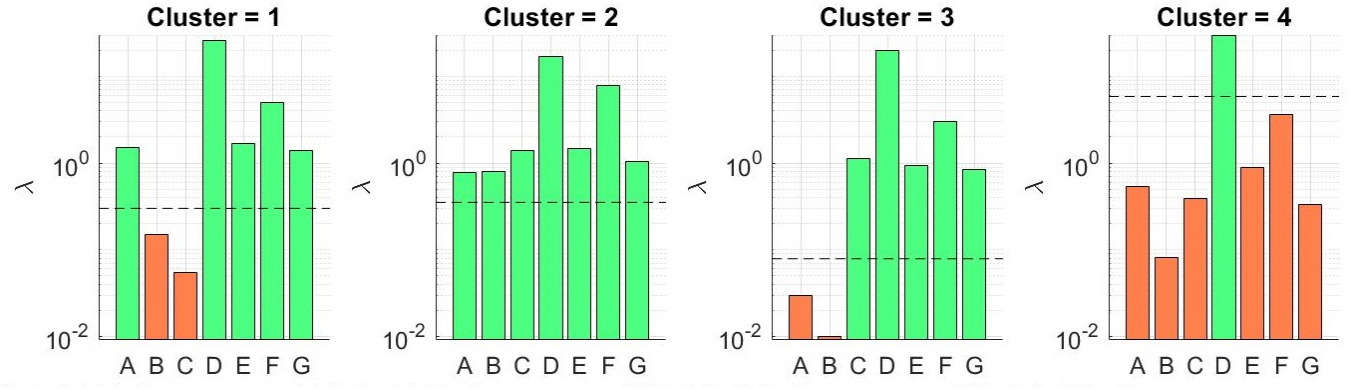
The new λ-levels for sensor selection for each cluster using the weighted group lasso method for $\delta_{h}=30$. A, B, C, D, E, F, and G represent environmental temperature, body temperature, humidity, EDA, HR, wrist accel, and ankle accel, respectively.

We then investigated the effect of using our weighted regularization method defined in Section 4.3. This method weights the feature groups in the lasso model according to the measured power usage of the sensor in the HET platform. Table 5 gives the updated current reductions using the weighted method. Figure 10 shows the λ-levels for sensor selection with the weighted method. Surprisingly, the weighted method actually used more sensors in some of the activity clusters. This was because, in our current wearable system, the most important sensors, namely the ankle accelerometer and EKG, are also the most power hungry sensors. This caused less penalization for other modalities. The ankle accelerometer and HR features now have lower λ-levels than in the normalized method. Therefore, when we penalized these sensors at a higher weight, the regularizer reduced their effect on the regression and thus required more sensors to maintain the optimal level of prediction accuracy. Prediction performance across all evaluation metrics for all clusters was not affected using the Weighted lasso approach.

## 6. Conclusions

We propose a methodology for power-optimized prediction of physiological signals from multi-modal data streams. We obtained promising preliminary results showing the benefit of applying our method to a wearable health monitoring platform. We found that utilizing an adaptive activity-aware sensor selection approach both reduces prediction error and increases power efficiency. Our results show that incorporating activity clustering has a positive impact on model performance and power efficiency as well.

We demonstrate the ability to personalize our model according to the specific user and application needs. For example, some applications may place higher priority on extended battery life and reliability than prediction accuracy. We demonstrate the trade-off between prediction accuracy and power efficiency by adjusting the regularizer term, λ, to balance the model according to the specific application requirements. We also investigated a weighted regularization approach which penalizes the sensors according to power usage.

As future work, we plan to extend this work by first testing our method on a much larger dataset including multiple subjects to further validate our preliminary results and account for variances in physiological responses between different subjects. The next step is to apply this technique to activities of daily life rather than to an exercise protocol in order to test performance on dynamic and randomly distributed activity states. We will also investigate how to reduce the error during transitional activity states utilizing time series features to capture the temporal behavior of the physiological signals. We plan to investigate improving prediction performance by incorporating our sensor selection method into time series models such as Recurrent Neural Networks.

## Figures and Tables

**Table 1:** HET Sensors with Sampling Rates.

Measurement	Platform	Measuring IC	Sampling Rate (Hz)
Accelerometer	Wrist and Chest	MMA7660 (NXP Semiconductors)	100
Electrocardiogram	Chestpatch	ADS1299 (Texas Instruments)	100
Temp./Humidity	Wristband	HDC1080 (Texas Instruments)	2

**Table 2:** HET average current measurements.

Wrist Sensor	Current (mA)	Chest Sensor	Current (mA)
Full Function	4.13	Full Function	5.89
No Accelerometer	3.81	No Accelerometer	3.19
No Environmental	3.86	No EKG	3.22

**Table 3:** Sensors included in the normalized model.

Sensor	$\delta_{h}=10$					$\delta_{h}=30$					$\delta_{h}=60$					$\delta_{h}=90$				
	$C_{1}$	$C_{2}$	$C_{3}$	$C_{4}$	NC	$C_{1}$	$C_{2}$	$C_{3}$	$C_{4}$	NC	$C_{1}$	$C_{2}$	$C_{3}$	$C_{4}$	NC	$C_{1}$	$C_{2}$	$C_{3}$	$C_{4}$	NC
Body Temp.																				
Env. Temp.																				
Humidity																				✓
Wrist Accelerometer	✓	✓	✓	✓	✓	✓	✓	✓	✓	✓	✓	✓	✓	✓	✓	✓	✓	✓	✓	✓
Ankle Accelerometer	✓	✓	✓	✓	✓	✓	✓	✓	✓	✓	✓	✓	✓	✓	✓	✓	✓	✓	✓	✓
EDA	✓	✓	✓		✓	✓	✓			✓	✓	✓	✓		✓	✓	✓	✓		✓
HR	✓	✓	✓		✓	✓	✓	✓		✓	✓	✓	✓		✓	✓	✓	✓		✓

**Table 4:** Current reduction using the sensor selection method on the HET in mA and percent of total power usage reduction for the different values of $\delta_{h}$. The last column corresponds to not using clustering.

$\delta_{h}$	$\Delta I_{c_{1}}$ (mA) (%)	$\Delta I_{c_{2}}$ (mA) (%)	$\Delta I_{c_{3}}$ (mA) (%)	$\Delta I_{c_{4}}$ (mA) (%)	$\Delta I_{\mathrm{avg}}$ (mA) (%)	$\Delta I_{\mathrm{NC}}$ (mA) (%)
10	0.838 (8.42)	0.838 (8.42)	0.838 (8.42)	3.823 (38.44)	1.009 (10.15)	0.838 (8.42)
30	0.838 (8.42)	0.838 (8.42)	1.15 (11.56)	3.823 (38.44)	1.047 (10.53)	0.838 (8.42)
60	0.838 (8.42)	0.838 (8.42)	0.838 (8.42)	3.823 (38.44)	0.995 (10.00)	0.838 (8.42)
90	0.838 (8.42)	0.838 (8.42)	0.838 (8.42)	3.823 (38.44)	1.001 (10.07)	0.575 (5.78)

**Table 5:** Current reduction using the power ratio weighted sensor selection method on the HET in mA and percent of total power usage reduction for the different values of $\delta_{h}$.

$\delta_{h}$	$\Delta I_{c_{1}}$ (mA) (%)	$\Delta I_{c_{2}}$ (mA) (%)	$\Delta I_{c_{3}}$ (mA) (%)	$\Delta I_{c_{4}}$ (mA) (%)	$\Delta I_{\mathrm{avg}}$ (mA) (%)	$\Delta I_{\mathrm{NC}}$ (mA) (%)
10	0.526 (5.29)	0.575 (5.78)	0.838 (8.43)	7.014 (70.53)	0.954 (9.59)	0.263 (2.64)
30	0.838 (8.43)	0	0.575 (5.78)	7.014 (70.53)	0.993 (9.98)	0.575 (5.78)
60	0.838 (8.43)	0	0.312 (3.14)	6.702 (67.39)	0.946 (9.51)	0.575 (5.78)
90	0.575 (5.78)	0.312 (3.14)	0.312 (3.14)	6.702 (67.39)	0.837 (8.37)	0.263 (2.64)
